# Decision-making for active living infrastructure in new communities: a qualitative study in England

**DOI:** 10.1093/pubmed/fdz105

**Published:** 2019-09-30

**Authors:** A Le Gouais, L Foley, D Ogilvie, C Guell

**Affiliations:** 1 MRC Epidemiology Unit, Centre for Diet and Activity Research (CEDAR), University of Cambridge, Cambridge CB2 0QQ, UK; 2 MRC Epidemiology Unit, University of Cambridge, Cambridge CB2 0QQ, UK; 3 European Centre for Environment and Human Health, University of Exeter Medical School, Truro TR1 3HD, UK

**Keywords:** management and policy, physical activity, places

## Abstract

**Background:**

Urban design can influence population levels of physical activity and subsequent health impacts. This qualitative study investigates local level decision-making for ‘active living’ infrastructure (ALI)—walking and cycling infrastructure and open spaces in new communities.

**Methods:**

Thirty-five semi-structured interviews with stakeholders, and limited ethnographic observations, were conducted with local government and private sector stakeholders including urban and transport planners, public health practitioners, elected councillors and developers. Interview transcripts were coded and analysed thematically.

**Results:**

Public health practitioners in local government could act as knowledge brokers and leaders to motivate non-health stakeholders such as urban and transport planners to consider health when designing and building new communities. They needed to engage at the earliest stages and be adequately resourced to build relationships across sectors, supporting non-health outcomes such as tackling congestion, which often had greater political traction. ‘Evidence’ for decision-making identified problems (going beyond health), informed solutions, and also justified decisions post hoc, although case study examples were not always convincing if not considered contextually relevant.

**Conclusion:**

We have developed a conceptual model with three factors needed to bridge the gap between evidence and ALI being built: influential public health practitioners; supportive policies in non-health sectors; and adequate resources.

## Introduction

The social determinants of health are shaped by policies and decisions in non-health sectors. National and international policies increasingly acknowledge the impact that the built environment can have on population health through physical activity,^1–3^ recognising the role that non-health sectors such as urban and transport planning can play in producing activity-promoting environments.^4^^,^^5^ Newly built communities can serve as ideal test sites for this public health strategy.

Evidence-based policy and decision-making is promoted within the health sector. However, urban designs are often locally developed by decisions-makers outside the health remit and broader concepts of ‘evidence’ than scientific research are involved.^6–9^ The role of scientific evidence in influencing policy and practice has been widely researched,^10–13^ but there remain limitations in understanding the facilitators and barriers to decision-making for healthy outcomes in traditionally non-health sectors.^14^ Communication and co-production of research are promoted to improve the relevance of evidence for uptake for better decision-making,^13^^,^^14^ but few studies have investigated the use of evidence, alongside other influences, at the local level.^15^^,^^16^

**Table 1.** Interview participant role in each local government area

**Role**	**Area 1**	**Area 2**	**Area 3**	**Total**
Councillors	1	1	1	3
Public health practitioners	1	1	1	3
Greenspaces stakeholders (including for parks, landscaping and footpaths)	2	1	2	5
Cycling stakeholders	2	0	2	4
Local authority (LA) urban planners	3	3	3	9
Private urban planners (including from master-planning developers and volume housebuilders)	4	2	1	7
LA transport planners	2	1	1	4
Private transport planners (contracted by master-planning developers)	1	0	1	2
Other (public sector, including police)	0	0	3	3
**Total**	**16**	**9**	**15**	**40**

In England, there is substantial political pressure to increase house building,^17^ and new communities with thousands of new homes are being built, designed and financed by developers (mostly from the private sector), guided by local planning policies. Decision-making for walking and cycling infrastructure and open spaces (‘Active Living Infrastructure’ (ALI)) in large developments ultimately lies with locally elected councillors, who grant planning permission. Local government urban planners are highly influential as they develop policy, negotiate with developers and advise councillors. Public health practitioners also work in local government, supporting the ‘health in all policies’^18^ agenda.

This study sought to understand how public health can influence decision-making for ALI in new communities. The research was guided by three main questions: (1) How does evidence, information or data influence decisions relating to ALI and what else is influential? (2) What leads to changes in plans of new residential developments or towns that affect walkability, cycling or open spaces? (3) What evidence or data could support more effective planning of ALI?

## Method

### Setting

Three local government areas of England (two unitary local authorities (LAs) and one with two-tier LAs: district and county) were purposively sampled, each with a large new housing development being planned and/or built (thousands of new homes plus local commercial centres). Settings included rural, peri-urban and urban areas with developments adjacent to existing urban areas, villages or involved urban regeneration. All three LAs were also chosen as they have a public health practitioner dedicated to urban planning, existing high levels of ALI, or both, and were therefore considered information-rich sample settings.^19^ The locations are not identified to ensure anonymity of study participants who come from small stakeholder groupings.

### Participants

Interview participants were purposively sampled across influential stakeholder groups for ALI. Snowball sampling of recommended knowledgeable expert stakeholders was conducted through initial contacts from local government and the private sector to arrive at a diverse sample of individuals from urban and transport planning, public health, environment, elected councillors, cycling groups and developers. In total, 40 stakeholders were interviewed during 35 interviews between October 2017 and June 2018 (Table 1). Limited ethnographic observations were also conducted during two urban planning meetings in two areas involving private sector developers, LA urban planners, public health practitioners, environment professionals and others to inform the analysis and aid triangulation.

### Data collection

Initial scoping discussions were conducted with 13 key stakeholders from the public and private sectors in transport, urban planning and public health (7 local government, 1 central government, 5 non-government). These helped with developing the interview guide (see supplementary data) to enable practitioner-relevant research.

Qualitative interviews were semi-structured and allowed flexibility to explore emerging issues. They aimed to understand how different stakeholders used evidence, information and data to influence decision-making for ALI (explained to participants as walking or cycling infrastructure or open spaces that could enable physical activity), and when and how they were involved in the planning and design process. We did not want to restrict definitions of ‘evidence’ and invited participants to interpret it as they saw fit. The topic guide was initially piloted with two participants to check relevance across different sectors (urban planning and public health). All interviewees provided written informed consent.

The 35 interviews were conducted by ALG either face-to-face (68% of participants, 81% of which were at the participants’ offices, the remainder at ALG’s office or a public café) or by telephone (32%) and took an average of 51 minutes each (range 21–97 minutes). All except one (at the participant’s request) were audio recorded and transcribed verbatim. Notes were made for the non-recorded interview, which were checked and edited by the participant. Field notes were made during ethnographic observations.

### Analysis

We conducted thematic analysis^19^ to allow for emergent, unanticipated issues to arise and to identify and analyse patterns in the data using a rigorous process of data familiarisation, coding and theme development.^20^ Interview transcripts and notes were coded by ALG and two interviews were coded independently by CG, supported by NVivo 12,^21^ allowing for reflection on and discussion of the codes. Theme development was conducted by ALG and iteratively discussed and revised with CG to develop the themes and interpretation.

## Results

Stakeholders used a variety of ‘evidence’ to influence designs of ALI: to identify a problem; inform solutions; or justify decisions post hoc (Table 2). Public health practitioners could be influential across non-health sectors. Barriers to ALI involved political, organisational and structural issues (Table 3).

### Problem and solution evidence

#### Evidence of a problem—needs assessment beyond health

Stakeholders were influenced indirectly by academic research, which informed national dialogue and organisational concern about levels of physical inactivity and health impacts. Participants generally understood that there is strong evidence of health benefits of physical activity, which they described as ‘common sense’. ‘Health Impact Assessments’^22^ conducted by developers were often not required in local planning policy or were reportedly weak due to lack of skills and enforcement mechanisms.

Overall stakeholders tended to prioritise more tangible ALI-related issues such as air quality, congestion and car parking. They used local (qualitative and quantitative) data extending beyond the health sector, for example combining local childhood obesity statistics with spatial data of quality assessment of parks or traffic congestion. Public opinion was also influential. Demonstrating local problems increased political motivation of councillors to act but restricted funding limited monitoring and the ability to use objectively measured data.

#### Evidence for a solution—knowing what works

Evidence for solutions to identified problems or needs was available within guidance material, based on academic evidence from evaluations and case studies, for example from Public Health England and the Town and Country Planning Association.^23^^,^^24^ This was particularly accessed by urban planners, developers and public health practitioners who understood the value of ALI for health and wanted workable solutions. However, some developers complained that health evidence struggled to reach non-health sectors and one transport planner described guidance for cycling infrastructure as ‘sporadic’ and ‘ad hoc’.

Public health practitioners were most likely to access research evidence, whereas councillors rarely did this, admitting it was difficult accessing information and, like other participants, often simply used internet search engines such as Google. A handful of LA and private urban planners had directly engaged with academics to create evidence of effectiveness of ALI through evaluating new housing developments, whilst some cycling stakeholders and police participants engaged with academics to increase their knowledge of best practice.

#### Retrospective evidence—justifying solutions already made

Sometimes health benefits of ALI were used to justify decisions post hoc. For example transport planners, who prioritised tackling congestion, acknowledged health benefits of walking and cycling infrastructure to support such investment over roads; developers justified spending on greenspaces to investors with research about impact on house prices,^25^ and sometimes used health evidence to justify less road construction, which was expensive, affecting profits.

### Resistance, power and relationships

#### Limitations of evidence

A lack of clear evidence of ALI impacts made it difficult for public health practitioners and developers to know what to promote. Urban planners focused on outputs rather than outcomes, for example that the construction of cycle routes was completed rather than whether routes would be well used. Councillors were reluctant to try new designs based on examples from other places, which did not appear contextually relevant, and were fearful of seemingly wasting resources on apparently ‘risky’ solutions, which could be politically damaging. This was particularly a problem where good-practice demanded a step change in quality from the status quo and opposition from car drivers or restricting house building were concerns. Developers were also reluctant to invest in walking and cycling infrastructure in areas with apparent low local demand because they did not believe it would increase house prices.

Economic effects of ALI were rarely considered because financial savings from health benefits of ALI did not directly affect local government budgets; therefore, many councillors were sceptical of its value. Also, cost–benefit analysis was difficult to use in the planning system because urban planners negotiate financial contributions from developers, without monetising potential benefits.

#### Influential individuals

Public health stakeholders could be influential, firstly as knowledge brokers sharing evidence about the health effects of ALI and providing practical solutions, but potentially also acting as leaders, building strong relationships to inspire decision-makers to raise up health in their consciousness and motivate them to argue for ALI. Where public health practitioners had a defined planning role, urban planners described them as ‘passionate’ and a ‘force of nature’ and participants explained that they broke down silos to motivate stakeholders across sectors, creating mutual benefits with other sectors’ outcomes, including air quality, noise, flooding, biodiversity, congestion, social cohesion, crime and house prices.

Urban planners met most regularly with developers and negotiated with multiple stakeholders who were said to push their own agendas. ALI could be difficult to achieve because of other demands and no defined minimum standards, but urban planners could influence designs if knowledgeable and motivated; however, they lacked specialist health understanding.

#### The value of early involvement

Most stakeholders understood that early engagement with developers, before planning applications were submitted, provided the greatest opportunity to influence ALI designs, and some were frustrated that LA urban planners involved them too late. It therefore appeared that LA urban planners needed to either understand the health impacts of a scheme themselves, which they struggled with, or be able to bring in other sources of knowledge and influence via public health practitioners.

### Barriers to innovation and change

#### Limited by policies

Stakeholders discussed a lack of national level standards and policies for ALI, which restricted quality. Participants said that local policies generally supported healthy developments but wording was vague without specifications for walking and cycling infrastructure and only quantities of open space required per population, not quality. Stakeholders described tensions between ALI and competing demands, including national planning and transport policies, which promoted house building,^26^ and transport assessment methods, which focused on road traffic analysis rather than ‘fluffy active travel stuff’ (LA transport planner). It seemed that local policies were important to set minimum standards for developments, which LA urban planners could then use to hold developers to account. Without defined policies, stakeholders said developers would only provide the minimum that they could get away with, unless they saw financial value in doing more.

Participants talked about difficulties in producing policies, which risked being unpopular to car drivers as councillors feared public backlash if congestion increased as a result of new development. So whilst some planners and developers wanted to be innovative, they were restricted by local policies, for example, specifying a minimum number of car parking spaces per house.

#### Watering down good designs

Even when ALI was initially well designed, participants described situations where plans could later change because minimum design standards were lacking—developers might try to reduce costs, plans were not enforced or concerns about crime led to watering down designs. Sometimes, the impracticality of plans became apparent too late, for example discovering that a football pitch was located on a slope, resulting in its purpose being changed.

Safety auditors often recommended changes to walking and cycling infrastructure because of safety concerns, and developers agreed to these changes to improve their chances of receiving planning permission and to ensure that the LA would take on long-term management of roads. Whilst public health practitioners also considered accident risks, they were more likely to take an holistic view. Finally, some participants were frustrated by schemes where walking and cycling routes were built after all houses were completed, apparently for cost reasons, because people then got ‘into bad habits’ (Greenspaces stakeholder) and therefore were less likely to use them.

**Fig. 1:**
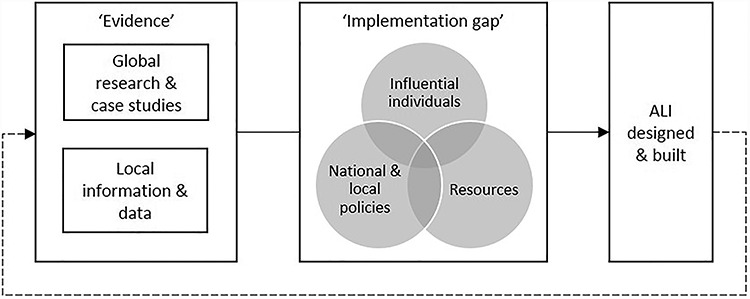
The ‘evidence-output implementation gap’.

#### Not enough resources

Most participants were concerned that LA urban planners were under resourced to engage with the right people, learn about best practice and ensure that health was adequately considered. Limited resources for monitoring and evaluation also restricted learning about effectiveness. Some stakeholders wanted to work more with public health, including master-planning developers, to get feedback on designs (in contrast to volume housebuilders whom participants said had no concern for health). However, most LAs in England did not have a public health practitioner dedicated to urban planning.

## Discussion

### Main findings of this study

We found that public health practitioners in local government could act as knowledge brokers and leaders, if engaged early enough, to motivate non-health stakeholders to consider health when designing and building new communities. ‘Evidence’ was found to be used to identify problems, inform solutions (noting that case study examples were often not considered contextually relevant) or justify decisions post hoc. However, it was influential public health practitioners who, if adequately resourced and with supportive policy environments, could share knowledge and inspire others not only to enable more ALI but also to ensure that it was attractive, convenient, safe and functional.^4^^,^^5^ This is summarised in Fig. 1 as an ‘evidence-output implementation gap’.

**Table 2.** Problem and solution evidence interview quotes

Many types of ‘evidence’	‘I think when we talk about evidence, I’m talking of a scale between anecdotal through to your proper published papers.’—Public health practitioner
Evidence of a problem—needs assessment beyond health	‘Air quality and congestion may be something that you could use more in terms of motivating them [politicians] to think a bit more differently in terms of modal shift, but I think the [physical] activity argument and the rest of it, I do not think that is as powerful to local councillors as the air quality issues are.’—LA urban planner ‘some [councillors] really need a very clear picture at a local level, before they’ll decide that it’s something they should be challenging the status quo on.’—LA urban planner
Evidence for a solution—knowing what works	‘I know there’s a lot of research and data being shared around that, that we are sort of desperate to get our hands on really because of probably things that we can be doing on that, I sort of think sometimes health is in danger of seeing itself as a sector that stays within its sector, rather than being part of transport and lifestyles and greenspace and built form and everything.’—Private urban planner ‘I do not think I am supplied, generally speaking, with as much evidence as I would like … recently there was the BMJ article, wasn’t there, on the health benefits of cycling earlier this year which I’ve been quoting very widely… I would like a bit more ammunition that I could use because cycleways you see are really really controversial, many motorists and of course most councillors are motorists, feel that cyclists get far too much money spent on them... it’s actually sometimes quite a struggle to persuade your colleagues that actually active modes deserve priority over road traffic.’—Councillor
Retrospective evidence—justifying solutions already made	‘if we need to justify the fact that we do spend quite a lot of money on greenspace we always feel quite comfortable that you can justify it because we have created an attractive space and actually the value of the homes is more than a development where you do not have a nice space around it … (and) the more you can do by cutting down trips by the way you design a place, and investing in public transport, then you do reduce your big spend on big bits of road … I do not think that is a driver, but it’s a way we then look to justify if anyone questions us as to why we are spending a lot of money on active neighbourhoods...’—Private urban planner
Limitations of evidence	‘what we are effectively doing is spending a lot of public money on the basis of a hunch here and a good idea there. Quite often things can be a good idea in one context, I think this is another thing that does not go on, which is actually contextualising the situation properly.’—Private urban planner ‘I think planning’s notorious, I mean the planning system can get you information on how many houses are built and whether they are occupied and whether the infrastructure that developers have to deliver is in, like have they built their roads …? Planning does not, planning kind of falls away a bit in terms of effectiveness when you are into places actually being used and lived in by people.’—LA Urban planner ‘if there is an example where it’s worked previously or it’s showing benefits and you can take any sceptic sort of person along and say, "Look, this is what we are going to do here" or you show a photograph of it, most people would be fine with that, but if, I think there is a reluctance to be the first to try something out in some ways.’—Greenspaces stakeholder ‘while I’m often told to look at what the Netherlands are doing and why can't we do that here, that’s not really much help... local evidence is better, if there were more of it it would be helpful.’—Councillor ‘enlightened members will care if it saves the NHS money, but many will say, “Well, that’s got nothing to do with us, that’s not part of our responsibility.”’—LA urban planner

**Table 3.** Power and relationships interview quotes

Influential individuals	‘For me, the data and evidence part is important but it’s also shaping it in the context of what the outcomes are for the other areas and departments and seeing it in that context as well and a lot of it is about building up the right relationships with the right people to be able to influence those developments and areas and programmes of work as well.’—Public health practitioner ‘You would not be able to achieve what we have achieved if you did not have people who were passionate about what they were doing and wanted to do things differently. I’ve worked in three local authorities and it’s quite easy for people to get into the tick box mentality. … I think when you have got passionate people who are committed to achieving a positive change in communities, it makes a real difference and it does not take a lot, it just takes a few people and they can have that ripple effect … in terms of improving longer term public health outcomes.’ LA urban planner ‘I am going into a meeting this afternoon with the promoters for [development], and I’m going to specifically ask them what are they doing in their master planning to allow for healthy lifestyles, so that’s something, me or the person who is in my [urban planning] job 5 years ago might not have asked specifically, and that is a direct result of public health coming into the councils … But I have only got … a little bit of understanding of all of the health outcomes that we might want to achieve …’ LA urban planner ‘because there is not a rule book that says for a new development you need to do this, then it’s individual people that then can make a difference or not … what arguments are you willing to have with developers and with colleagues to an extent, you know, you do not necessarily have a consensus within an organisation about what infrastructure’s needed, how it should be designed, what it should look like, how are people going to use it...’—LA transport planner
The value of early involvement	‘we are brought into it too late in the planning stage … I think if we were brought in at the stage earlier our options would be bigger, we’d have more options to do something innovative.’—Other ‘the ultimate aim should be that we shape the scheme earlier before it gets to application because once it’s got to application there’s only so much you can influence at that stage whereas when it’s in a design stage and in the pre-planning stage that’s where you have the greatest influence.’—Public health practitioner
Limited by policies	‘[LA] Planning teams, they can be very good enablers and they can be very supportive, but they are only supportive if the local plan has the right policies in that they can then fight.’—LA urban planner ‘if you are going to say that you want to shift the mode of travel to cycling and walking and have a real dramatic change, you have got to have a dramatic policy change to enable that to happen.’—Public health practitioner ‘We are given parameters to work to, that’s what we work to. If we are going to go overboard and provide more than what is required, it’s because we think it adds more value to our bottom line, yeah, but otherwise we just stick to what we are told we need to do.’—Private urban planner
Watering down good designs	‘quite often some developers will make promises in an outline planning consent, but by the time it comes to delivering stuff on the ground other hidden costs have emerged, which they did not foresee, and then perhaps certain pieces of, you know, fairly important walk cycle infrastructure get watered down or removed…’—Private transport planner ‘it tends to be that Road Safety have the final say on everything, which is not always to the benefit of cycling and walking, and in actual fact sometimes to the disadvantage of cycling and walking, because we’ll have created a nice little shared use route to modern design standards and gives priority to cyclists and walkers and is all lovely and ideal, and perfect in a perfect world for active travel, and Road Safety come along and say, "no you cannot do that, it’s dangerous" … Road Safety trump every scheme, every time.’—LA transport planner
Not enough resources	‘[LA] Planning Teams can be a barrier if they are under pressure, so if they are under pressure to get an application turned round in the 8 weeks then all the ‘nice to do’ stuff that I want to see in, gets dropped, all the other bits and pieces that we would fight for becomes that much harder to fight for, so the Planning Team is key, because they are the ones that make the ultimate recommendations to the Planning Committee to approve or not approve … sometimes they get so bombarded with all the applications coming through they do not really have that time to sit down and do all the pre-app meetings and bring in everyone that needs to be.’—Public health practitioner ‘I’d like to work with [public health] more but I do not seem to get an answer all the time … like most departments, they have restructured, reduced their services’—Cycling stakeholder ‘So, typically, you know, on a lot of developments we are involved with, there is not a health person, in inverted commas, who you can speak to at a local authority to sort of say, “Well, how do you think this master plan is shaping up?”’—Private urban planner

### What is already known on this topic

Findings about the types of evidence used reflect previous studies: scientific evidence hierarchies are unlikely to be considered in non-health disciplines,^8^^,^^27^ and local evidence of effectiveness and public opinion is highly valued,^15^ often for broad outcomes of interest including congestion and air quality; if academic research is used, then its external validity is important in determining whether a solution is applicable to decision-makers’ local contexts.^6^ There are demands for improving the quality of evidence around effectiveness of ALI for population physical activity,^4^^,^^5^ which could be supported by wider monitoring and evaluation in LAs. A lack of research in this area has been explained previously as an ‘inverse evidence law’^28^ whereby the least amount is known about interventions which are most likely to influence whole populations, and previous research has highlighted challenges in creating evidence to inform practice.^29^

### What this study adds

Knowledge exchange literature advocates for knowledge brokers to translate research into policy and practice, enabling joint working for mutually beneficial outcomes and ‘learning to speak the same language’.^30–33^ We found that public health practitioners in local government can adopt knowledge broker roles to promote ALI. However, scientific evidence alone is insufficient to influence policy and practice in local government^34^ and political feasibility must be considered.^8^ Research has demonstrated decision-making to be non-linear and influenced by multiple factors.^30^ This study also echoes findings from policy theory, recognising the importance of actors, institutions, networks, ideas/beliefs, policy context and events,^35^ and specifically relationships and leadership in local government.^9^^,^^14^ Kingdon described three streams of problem, policy and politics that needed to coincide to provide a ‘window of opportunity’ for change^36^ and a similar analogy was seen for decision-making in this study: problem ‘evidence’ needs to be identified, policies and solutions made available and politics supportive (aided by influential individuals) for healthy ALI. The advocacy coalition framework^37^ also shares relevance with our findings, particularly for cycling infrastructure where opposing ‘coalitions’ of pro- and anti-cycling groups can be at loggerheads. Central to Kingdon’s framework is the ‘policy entrepreneur’ to instigate change, echoed in our study in a role shared between urban planners acting as negotiators and public health practitioners acting as knowledge brokers and charismatic leaders.^38^ Further understanding is needed about the nuances underlying these ‘broker’, ‘champion’ or ‘policy entrepreneur’ roles, and what makes them influential or effective to practice the ‘art’, not only the science, of public health.^39^

We developed a conceptual model with three factors needed to fill the ‘evidence-output implementation gap’ (Fig. 1) for ‘evidence’ to support ALI: influential individuals such as public health practitioners in local government who can engage early with developers to improve designs and avoid later dilution; national and local urban planning and transport sector policies and standards which enable ALI; and adequate resources for collaborative working and learning.

This study highlighted a lack of contextually specific examples available to local decision-makers, which reduced political acceptability of change for ALI. Although complex interventions will not follow a formula,^8^ examples from similar places are more persuasive to local level decision-makers. Figure 1 includes a dotted line to show a translational framework approach,^40^ where greater monitoring and evaluation of ALI at scale could strengthen the evidence-base. This requires motivational leadership and collaboration across LAs to change attitudes and emphasise effectiveness of ALI outcomes over potentially ineffectual outputs.

### Limitations of this study

LAs are heterogeneous and focusing on three areas of England may have missed insights from other contexts. ALG has a background in public health, civil engineering and local government, which helped to build rapport with many study participants. However, participants came from many sectors; therefore, ALG had less experience in some areas. Snowball sampling following the recommendation of key stakeholders might have led to likeminded participants, but it enabled access to important stakeholders, some of whom were unanticipated. New communities were at different stages of development, but limited timeframes meant it was not feasible to follow decision-making through from conception to construction.

## Conclusion

Public health practitioners can help bridge the ‘evidence-output implementation gap’ for quality ALI, if engaged early, acting as influential knowledge brokers and leaders to motivate non-health stakeholders, such as urban and transport planners. Supportive policies, greater resourcing and increased monitoring for contextually relevant examples would also help.

## Supplementary Material

suppl_data_fdz105

## Notes

**A. Le Gouais**, PhD Researcher

**L. Foley**, Senior Research Associate

**D. Ogilvie**, Programme Leader

**C. Guell**, Lecturer
