# Supplementary material for: Decision-making for active living infrastructure in new communities: a qualitative study in England
Source: J Public Health (Oxf). 2019 Sep 30;42(3):e249–58. doi: 10.1093/pubmed/fdz105 (PMC7435215; doi:10.1093/pubmed/fdz105)
Supplement: suppl_data_fdz105 [file suppl_data_fdz105.zip › COREQChecklist-Decision-making for ALI.docx]

**Consolidated criteria for reporting qualitative studies (COREQ): 32-item checklist**

Developed from:

Tong A, Sainsbury P, Craig J. Consolidated criteria for reporting qualitative research (COREQ): a 32-item checklist for interviews and focus groups. *International Journal for Quality in Health Care*. 2007. Volume 19, Number 6: pp. 349 – 357

**Manuscript entitled "Decision-making for active living infrastructure in new communities: a qualitative study in England"**

| **No. Item** | **Guide questions/description** | **Reported on Page #** |
| --- | --- | --- |
| **Domain 1: Research team and reﬂexivity** |  |  |
| *Personal Characteristics* |  |  |
| 1. Interviewer/facilitator | Which author/s conducted the interview or focus group? | ALG conducted the participant interviews. Page 4 |
| 2. Credentials | What were the researcher’s credentials? E.g. PhD, MD | ALG has MEng in Engineering, Economics & Management, MSc in Water Management. |
| 3. Occupation | What was their occupation at the time of the study? | ALG was PhD student at CEDAR. |
| 4. Gender | Was the researcher male or female? | ALG is female. |
| 5. Experience and training | What experience or training did the researcher have? | ALG has training in interviewing and data analysis; she was supervised by CG [as well as DO and LF] who has over 10 years of extensive research experience and teaches qualitative research methods |
| *Relationship with participants* |  |  |
| 6. Relationship established | Was a relationship established prior to study commencement? | Some participants were involved in the scoping discussions with ALG prior to interview. She met some other interview participants at one of the events where ethnographic observation occurred, prior to interview. Page 4 |
| 7. Participant knowledge of the interviewer | What did the participants know about the researcher? e.g. personal goals, reasons for doing the research | Some information was provided in Participant Information Sheets outlining the purpose of the study and introducing ALG. |
| 8. Interviewer characteristics | What characteristics were reported about the interviewer/facilitator? e.g. Bias, assumptions, reasons and interests in the research topic | Limitations are included in the discussion section including ALG’s background and the varied sectors that participants came from. Page 10 |
| **Domain 2: study design** |  |  |
| *Theoretical framework* |  |  |
| 9. Methodological orientation and Theory | What methodological orientation was stated to underpin the study? e.g. grounded theory, discourse analysis, ethnography, phenomenology, content analysis | A qualitative pragmatic study using thematic content analysis to explore experiences of decision-making. Page 5 |
| *Participant selection* |  |  |
| 10. Sampling | How were participants selected? e.g. purposive, convenience, consecutive, snowball | Snowball sampling of key stakeholders in purposively selected local government areas.  Page 4 |
| 11. Method of approach | How were participants approached? e.g. face-to-face, telephone, mail, email | Potential participants were approached by email by ALG. Page 4 |
| 12. Sample size | How many participants were in the study? | 40 interview participants. Page 4 |
| 13. Non-participation | How many people refused to participate or dropped out? Reasons? | Potential participants were invited by email. Some did not reply and no reason was given. |
| *Setting* |  |  |
| 14. Setting of data collection | Where was the data collected? e.g. home, clinic, workplace | Study participants’ offices (55%); ALG’s office (8%); public café (5%); Telephone (33%). Page 4 |
| 15. Presence of non-participants | Was anyone else present besides the participants and researchers? | No one else present. |
| 16. Description of sample | What are the important characteristics of the sample? e.g. demographic data, date | Sample characteristics are described in table 1. |
| *Data collection* |  |  |
| 17. Interview guide | Were questions, prompts, guides provided by the authors? Was it pilot tested? | Interview guide provided in supplementary material. Piloted and minor edits made. Page 4 |
| 18. Repeat interviews | Were repeat interviews carried out? If yes, how many? | No repeat interviews. |
| 19. Audio/visual recording | Did the research use audio or visual recording to collect the data? | Audio-recordings of interviews for all except one. Page 4 |
| 20. Field notes | Were ﬁeld notes made during and/or after the interview or focus group? | Brief notes were taken during the audio-recoded interviews; notes made for the non-recoded interview; field notes made for ethnographic observation. Page 4/5 |
| 21. Duration | What was the duration of the interviews or focus group? | Interviews lasted an average of 51 minutes each (range 21 – 97 minutes). Page 4 |
| 22. Data saturation | Was data saturation discussed? | Saturation was not sought for this study as it focussed on key stakeholders in purposively selected areas. |
| 23. Transcripts returned | Were transcripts returned to participants for comment and/or correction? | This was not routinely done, however one participant requested to have their transcript returned. They did not provide any feedback. |
| **Domain 3: analysis and ﬁndings** |  |  |
| *Data analysis* |  |  |
| 24. Number of data coders | How many data coders coded the data? | ALG coded all data; CG coded two interviews independently. Page 5 |
| 25. Description of the coding tree | Did authors provide a description of the coding tree? | Corresponding author can be contacted for the coding tree. |
| 26. Derivation of themes | Were themes identiﬁed in advance or derived from the data? | Coding was directed by research objectives but themes derived inductively from the data. Page 5 |
| 27. Software | What software, if applicable, was used to manage the data? | NVivo 12. Page 5 |
| 28. Participant checking | Did participants provide feedback on the ﬁndings? | No, although this is planned for future research |
| *Reporting* |  |  |
| 29. Quotations presented | Were participant quotations presented to illustrate the themes/ﬁndings? Was each quotation identiﬁed? e.g. participant number | Quotations presented to illustrate the themes in tables 2 and 3. Participants identified by their role. |
| 30. Data and ﬁndings consistent | Was there consistency between the data presented and the ﬁndings? | Yes, there is consistency between data and the findings. |
| 31. Clarity of major themes | Were major themes clearly presented in the ﬁndings? | Yes, major themes are clearly presented in the findings. |
| 32. Clarity of minor themes | Is there a description of diverse cases or discussion of minor themes? | Yes, minor themes and diverse cases were described and discussed where they occurred. e.g. Results, Page 5-8 |
