# Supplementary material for: Decision-making for active living infrastructure in new communities: a qualitative study in England
Source: J Public Health (Oxf). 2019 Sep 30;42(3):e249–58. doi: 10.1093/pubmed/fdz105 (PMC7435215; doi:10.1093/pubmed/fdz105)
Supplement: suppl_data_fdz105 [file suppl_data_fdz105.zip › Interview guide - Decision-making for ALI.docx]

|  | **Interview guide** | |
| --- | --- | --- |
| **#** | **Main Questions** | **Possible follow-up/prompts** |
| **Background to involvement and personal attitude to healthy infrastructure:** | | |
| 1 | Can you outline **your role**? | Can you describe how and when you are involved with planning or developing walking and cycling infrastructure and open spaces?  What type of developments are you normally involved with? |
| 2 | What is your view on the walking and cycling infrastructure, and open spaces, in this areas? | How **walkable** or **cycleable** are developments/ this development?  How do you feel about the amount and quality of **open space**?  Why are there **differences** between different areas? |
| **Knowledge exchange and evidence influencing the decision-making process:** | | |
| 3 | Can you tell me about the **sources of information, knowledge or data** that you use to help you make or influence decisions? | Where do you go for **information**? What are the key **guidance documents?**  How useful is evidence from **other settings**? Is **National guidance** useful?  What other **evidence** is used to inform **decision-making**? |
| 4 | What is your view of **economic analysis** for healthy infrastructure? | Is **cost effectiveness** useful?  Do you think savings to the **NHS** can influence infrastructure decisions?  Do you assess **value for money**?  *Developer*: How can **viability** impact on levels of open space? Could it affect walking and cycling routes? |
| 5 | Do you do or use **monitoring** or **effectiveness** data for walking or cycling? | How **useful** is monitoring data? |
| 6 | What **other information** or **evidence** would be useful to you? | Are there times where your argument would have been strengthened by better information or evidence? |
| 7 | Can you tell me about your view of **HIAs**? | What are the **benefits or problems** with HIA?  Can planning be **refused** on health grounds? |
| **Key stakeholders and their relationships** | | |
| 8 | Who are the main **supporters and opponents** to planning healthy infrastructure? | Can you describe the type of working **relationship** between stakeholders (e.g. planners, public health, developers, councillors etc.). Are they **collaborative**? **Silos**? |
| 9 | What do you think is the **main driver** for building active infrastructure? | Congestion, cost, health, etc.? |
| 10 | What do you think is needed to help **you** to enable more healthy infrastructure to be built? | What **limits** your influence e.g. time, budget, silos, politics, interest, relevance etc.? |
| 11 | What do you think **motivates others** to support walkable and cycling developments? | e.g. Traffic, the economy, politics, congestion, safety, carbon footprint, air pollution, community cohesion, local economy, economic benefits etc.? |
| 12 | Do you feel encouraged to be **innovative** and try new approaches? | What **supports** or **inhibits** innovation and trying new things?  Can you comment on the level of influence by **central government**? |
| **Drivers for change during the planning process** | | |
| 13 | Can you explain to me **how and when changes can occur** in development plans, particularly related to active living infrastructure? | Do you have a recent **example**?  **What happened and why?**  **Who** was involved? |
| **Changes in healthy planning over time** | | |
| 14 | Have you seen any **changes** to how planning decisions account for health over the last few years, since public health moved into local authorities in 2013? | What other things have **influenced** how public health issues are considered in the last few years? |
| Thank you very much for answering my questions. Is there anything else that you would like to say on this topic? **Comments**? **Feedback**? | | |
